# Study protocol for Near-infrared molecular imaging for lung cancer detection and treatment during mini-invasive surgery (phase II Trial) - (the RECOGNISE study)

**DOI:** 10.1186/s12885-024-12859-6

**Published:** 2024-09-02

**Authors:** Eleonora Della Beffa, Paraskevas Lyberis, Giulio Luca Rosboch, Alberto Arezzo, Filippo Lococo, Laura Carena, Elisa Sciorsci, Valentina Monica, Paolo Olivo Lausi, Veronica Dusi, Francesco Paolo Busardò, Elena Buffa, Rachele Stefania, Giovannino Ciccone, Chiara Monagheddu, Beatrice Maria Capello, Raffaella Vancheri, Pamela Garrone, Fulvio Gabbarini, Francesco Cattel, Enrico Ruffini, Francesco Guerrera

**Affiliations:** 1 Department of Cardio-Thoracic And Vascular Surgery, Azienda Ospedaliera Universitaria Città della Salute e della Scienza di Torino, Corso Dogliotti, 14, Torino, 10,126 Italy; 2 Department of Surgical Science, University of Torino, Corso Dogliotti, 14, Torino, 10,126 Italy; 3 Department of Anaesthesia, Intensive Care and Emergency, Azienda Ospedaliera Universitaria Città della Salute e della Scienza di Torino, Torino, Italy; 4 IRCCS-Fondazione Policlinico Gemelli, Università Cattolica del sacro Cuore, Rome, Italia; 5 S.C. Farmacia Ospedaliera, AOU Città della Salute e della Scienza di Torino, Torino, Italy; 6 Candiolo Cancer Institute, FPO-IRCCS, Candiolo (Torino), Torino, Italy; 7 Department of Oncology, University of Torino, Torino, Italy; 8 Division of Cardiology, Department of Medical Sciences, University of Turin, Torino, Italy; 9 Department of Science and Technological Innovation, University of Eastern Piedmont “Amedeo Avogadro”, Alessandria, Italy; 10 Unit of Clinical Epidemiology, AOU Città della Salute e della Scienza di Torino and CPO Piemonte, Torino, Italy; 11 Pediatric Cardiology Division, Children Hospital Regina Margherita, Torino, Italy; 12 Department of Internal Medicine, University of Turin, Torino, Italy

**Keywords:** Cetuximab-IRDye800CW, Lung cancer, VATS, Thoracic surgery, Lung nodule, NIR imaging, Fluorescence

## Abstract

Introduction. To date, radical surgery remains the best curative option in patients with early-stage lung cancer. In patients with small lung lesions, video-assisted thoracic surgery (VATS) should be increasingly chosen as a fundamental alternative to thoracotomy as it is associated with less postoperative pain and better quality of life. This scenario necessarily increases the need for thoracic surgeons to implement new localization techniques. The conventional near-infrared (NIR) indocyanine green (ICG) method demonstrated a significant limitation in deep cancer recognition, principally due to its intrinsic low-depth tissue penetration. Similarly, the lymph-node sentinel approach conducted by the ICG method was demonstrated to be inefficient, mainly due to the non-specificity of the tracker and the irregular pathway of pulmonary lymph node drainage. Our study aims to evaluate the effectiveness of Cetuximab- IRDye800CW in marking lung nodules and mediastinal lymph nodes. Methods and analysis. This study is defined as an open-label, single-arm, single-stage phase II trial evaluating the effectiveness of Cetuximab-IRDye800CW in detecting tumors and lymph-node metastases in patients with lung cancer who are undergoing video-assisted thoracic surgery (VATS). Cetuximab is a monoclonal antibody that binds, inhibits, and degrade the EGFR. The IRDye^®^ 800CW, an indocyanine-type NIR fluorophore, demonstrated enhanced tissue penetration compared to other NIR dyes. The combination with the clinical approved monoclonal antibody anti-epidermal growth factor EGFR Cetuximab (Cetuximab-IRDye800) has shown promising results as a specific tracker in different cancer types (i.e., brain, pancreas, head, and neck). The study’s primary outcome is focused on the proportion of patients with lung nodules detected during surgery using an NIR camera. The secondary outcomes include a broad spectrum of items, including the proportion of patients with detection of unexpected cancer localization during surgery by NIR camera and the proportion of patients with negative surgical margins, the evaluation of the time spawns between the insertion of the NIR camera and the visualization of the nodule and the possible morbidity of the drug assessed during and after the drug infusion. Ethics and dissemination. This trial has been approved by the Ethical Committee of Azienda Ospedaliera Universitaria Città della Salute e della Scienza di Torino (Torino, Italy) and by the Italian Medicines Agency (AIFA). Findings will be written as methodology papers for conference presentations and published in peer-reviewed journals. The Azienda Ospedaliera Universitaria Città della Salute e della Scienza di Torino, the University of Torino, and the AIRC Public Engagement Divisions will help identify how best to publicize the findings.

**Trial registration** EudraCT 202,100,645,430. ClinicalTrials.gov NCT06101394 (October 23, 2023).

## Background

Lung cancer is the leading cause of cancer deaths in the European Union (EU) (267,000 deaths/year) and the fourth most common cancer (321,000 new cases/year) [1]. To date, radical surgery remains the best curative option in patients with early-stage lung cancer. Moreover, cancer screening programs have led to frequent diagnoses of indeterminate lung lesions, many of which require surgical biopsy for diagnosis and intervention [2]. For example, available data showed that 5.9% of the European population over 15 years of age consumed at least 20 cigarettes per day (8.4% in the male population), and around 12.6% consumed less than 20. The recent lung cancer screening studies documented a prevalence of indeterminate pulmonary nodules as high as 50% in high-risk smokers, with a cancer detection rate in the overall screened population of 1% [3]. Fascinatingly, 69% of screen-detected lung cancers were detected at early-stage IA or IB [2]. Finally, studies on lung cancer screening, like the NELSON trial, show a 26% reduction in lung cancer deaths at ten years. The potential social impact of the present project is linked to establishing this screening program in Europe. One could estimate that if applied in the high-risk European population (i.e., high smokers), the screening could identify 3.5 million indeterminate pulmonary nodules, of which 250.000 are early-stage lung cancer [2]. Therefore, the use of the NIR-tracker we propose could consent to the use of minimally invasive surgery in this scenario as a diagnostic and therapeutic procedure. Consequently, it could determine the reduction of time to diagnosis, morbidity, mortality, and costs of postoperative care associated with more invasive surgical procedures.

To date, lung anatomical resection with lymphadenectomy remains the best curative option in patients with early-stage non-small cell lung cancer (NSCLC) [4]. Moreover, cancer screening programs have led to frequent diagnoses of indeterminate lungs, many of which require surgical biopsy for diagnosis and intervention [2]. Nevertheless, the increase in the utilization of minimally invasive procedures (e.g., video-assisted thoracic surgery -VATS- and robotic-assisted thoracic surgery -RATS-) remains mandatory in order to reduce the significant morbidity of classic surgery, the surgical trauma, to preserve organ’s function and to improve patient’s quality of life [5]. Nevertheless, minimally invasive surgery represents the surgical approach of choice in less than 40% of lung anatomical resections conducted in Europe [6]. One of the significant issues that hinder the application of VATS and RATS to most early-stage NSCLC patients is the difficulty in recognizing lung nodules located deep in the lung parenchyma and, consequently, not visible with the traditional camera system [7]. Indeed, VATS and RATS do not consent to manual lung palpation, making localizing the not superficial lung nodule problematic. Several approaches have been developed to enhance the localization of indeterminate lung nodules and decrease the time to diagnosis and rate of conversion to open surgery. Nevertheless, none of these are 100% sensitive or without complications, and they also have severe grades [8].

Numerous preoperative methods are being employed, including percutaneous CT-guided placement techniques, encompassing the use of micro-coils, hook-wires, and spiral-wires [8, 9]. These devices can support nodule localization during minimally invasive lung procedures; nevertheless, they could be easily displaced during patient transport and positioning, intraoperative atelectasis, single lung ventilation, and surgeon manipulation. Moreover, some locations of the lungs, such as the apex, near the diaphragm, and in the proximity of the mediastinum and great vessels. Furthermore, all these preoperative localization techniques require two different procedures, one for the CT-guided referral placement and one for surgical treatment. Finally, the rate of pneumothorax, hemorrhage, and subcutaneous emphysema are not insignificant, and these complications are mandatory to avoid in several sub-groups of patients. Other pre-operative methods encompass the use of dye marking by methylene blue or fluorescent [8]. Nevertheless, the accuracy of the staining of the targeted area is greatly affected by the time between tumor marking and thoracoscopy. In particular, the significant impact is on the difficulty of dye visualization during operation, limited information on lesion depth, and rapid diffusion of dye into the surrounding lung parenchyma between the time of injection and surgery. Of note, methylene blue has limited application in patients with anthracotic pigmentation. Moreover, these techniques require two procedures for diagnosis and treatment. Lastly, these procedures remain complicated by the risk of pneumothorax, bleeding, dye air embolism, cerebrovascular accident, and cases of lethal anaphylaxis to the dye of choice.

On the other hand, clinical pre-operative staging and surgical planning are based on preoperative images taken before surgery, either by computed tomography (CT), positron emission tomography (PET), or magnetic resonance imaging (MRI). These preoperative imaging assessments frequently underestimate lymph node involvement and secondary localizations. This results in an upstaging after surgical resection ranging from 9 to 24% in clinical Stage I lung cancer [10, 11]. Nevertheless, based on the direct injection of a tracker in the principal tumoral mass, the current intraoperative imaging system demonstrated a substantial limitation in lung cancer. This is principally due to the deeper location of the lymph nodes, usually profoundly engaged in normal fat tissue, and to irregular lymph node drain system in the respiratory region [12].

In this context, intraoperative fluorescence imaging can enhance the real-time identification of cancer cells during minimally invasive surgical procedures. This could overcome the difficulty of finding cancer nodules located deep in the lung parenchyma, not visible on the surface of normal, uninvolved tissue. The Near-infrared (NIR) fluorescence (700–1,000 nm) detection avoids biomolecules’ natural background fluorescence interference, which provides high contrast between the target and background tissues in small animals. NIR fluorophores have a more comprehensive dynamic range and minimal background fluorescence because of reduced scattering compared with visible fluorescence detection. However, the conventional near-infrared (NIR) indocyanine green (ICG) method demonstrated a significant limitation in deep cancer recognition, principally due to its intrinsic low-depth tissue penetration [13]. Similarly, the lymph-node sentinel approach conducted by the ICG method was demonstrated to be inefficient, mainly due to the non-specificity of the tracker and the irregular pathway of pulmonary lymph node drainage [12, 14].

The IRDye^®^ 800CW is an indocyanine-type NIR fluorophore with peak absorption at 775 nm and peak excitation emission at 796 nm. It provides a quantum yield of 9% and an extinction coefficient of 242,000 M-1 cm-1. Compared to other NIR dyes, the IRDye^®^ 800CW demonstrated enhanced tissue penetration [15].

Epidermal growth factor (EGF) is a 53–amino acid cytokine (6.2 kDa) that is secreted by ectodermic cells, monocytes, kidneys, and duodenal glands. EGF stimulates the growth of epidermal and epithelial cells. EGF, at least seven other growth factors, and their transmembrane receptor kinases play essential roles in cell proliferation, invasion, metastasis, neovascularization, adhesion, migration, differentiation, and inhibition of apoptosis. The EGF receptor (EGFR) family consists of four transmembrane receptors, which include EGFR (HER1/erbB-1), HER2 (erbB-2/neu), HER3 (erbB-3), and HER4 (erbB-4); and is commonly overexpressed in lung cancer [16]. Cetuximab is a monoclonal antibody that is able to inhibit and degrade the EGFR. Given intravenous infusion (IV), Cetuximab binds to the EGFR and stops the binding and activation of the downstream signaling pathways. Moreover, as we previously published, EGFR mutation is linked with skip-metastasis phenomena (i.e., pathologically proved mediastinal lymph node involvement in the absence of intrapulmonary or hilar lymph node disease) [17].

The IRDye800 conjugated with the clinically approved monoclonal antibody anti-epidermal growth factor EGFR Cetuximab (cetuximab-IRDye800) has shown promising results as a specific tracker in other cancer types (i.e., brain, pancreas, head, and neck) [18–20].

In detail, Cetuximab was incubated in the dark at room temperature with IRDye 800CW NHS Ester (LI-COR, USA) in 0,1 M potassium phosphate buffer (pH 7.8) for 2 h. The unconjugated dye was removed by size exclusion chromatography, using 0,9% NaCl as eluent, on an Amersham Biosciences AKTA FPLC System. The dye concentration was measured by absorption at 774 to confirm the number of fluorophore molecules conjugated to each mAb.

## Design

This study is defined as an open-label, single-arm, single-stage phase II trial evaluating the effectiveness of cetuximab- IRDye800 in detecting tumors and lymph-node metastases in patients with lung cancer who are undergoing minimally invasive thoracic surgery.

The trial has been registered to ClinicalTrial.gov with the registration number NCT06101394.

*Ethical committee referral number: 00136/2022*

## Methods

### Materials and equipment

We included patients diagnosed with NSCLC in clinical stage I (according to 8th Edition of TNM Staging System) who have been considered candidates for minimally invasive surgical resection after pre-operative assessment, including chest CT scans, PET, and respiratory function tests.

Regarding the age, we included patients ranging from 18 to 80. Patients with adequate bone marrow, kidney, liver, and heart function were included in the study. The included patients must have a PS ≤ 2, and potentially fertile female subjects must agree to use highly effective contraception throughout the study and for three months after the last dose of the study medication (According to CTFG recommendations related to contraception and pregnancy testing in clinical trials - Version 1.1–2020). The patients excluded from the study presented with characteristics like previous systemic treatments (chemotherapy, immunotherapy) for lung cancer, prior radiotherapy on lung or mediastinum, concomitant disorders that compromise the ability to adhere to the procedures of the protocol, Hb levels < 9 gm/dL, platelet count < 100,000/mm3, leukocyte count < 3000/mm3, absolute neutrophil count < 1500/mm3, electrolytes lower than the lower limit of normal per institution average lab values, TSH < 13 micro international units/mL, administration of an investigational drug within 30 days before the first dose of cetuximab IRDye800, MI, UA, heart failure, stroke or liver failure within 6 months before enrollment, previous infusional reactions to cetuximab or other monoclonal antibody therapies, evidence of QT prolongation, current therapy with antiarrhythmic agents including quinidine, procainamide, dofetilide, amiodarone, sotalol. Both pregnancy and present breastfeeding were considered exclusion criteria.

The study’s primary outcome is focused on the proportion of patients with lung nodules detected during surgery by NIR camera. The objective is to evaluate if cetuximab-IRDye800 can make visible lung nodules to the NIR camera through surgical procedures. The NIR camera used will be the EleVision™-Iridium VS3 (Medtronic, Dublin, Ireland). The detection of lung nodule NIR emission is registered in the first 5 min of the NIR camera activation during the surgery. The nature of the identified nodules (neoplastic or not) will be recorded during the surgery (sending the resected specimen for intraoperative pathology report (i.e., frozen section), later confirmed by the definitive pathology report) or 20–30 days after surgery according to the standard procedures in our center for the pathologic analysis of the operative specimens.

The secondary outcomes include a broad spectrum of items: the proportion of patients with unexpected cancer localization detected during surgery by NIR camera, the proportion of patients with negative surgical margins, the evaluation of the time spawns between the insertion of the NIR camera and the visualization of the nodule; and the possible morbidity of the drug assessed during and after the drug infusion.

The data will be collected from patient’s clinical records, entered in a secured password-locked web application, and then organized in a second password-locked database for easier data management. The data will include, at each relevant time point (Table 1), patient identification and anonymous demographic data, comorbidities, concomitant medications, vital signs, height and weight, Performance Status, adverse events (date of onset and resolution, maximum grade, relation to the study drugs), laboratory results, target, and non-target lesions assessments (dates and results), pulmonary function test, surgical details, post-operative data (complications, pain evaluate by NRS scale system, hospital length of stay) and final pathological staging. After the end of the protocol treatment, patients will be followed according to clinical practice (i.e. outpatient clinic follow-up visit with thorax and abdomen CT scans).

The expected rate of subjects lost to follow-up after surgery for early-stage lung cancer (as documented by large randomized clinical trials in this setting) is likely less than 5% and should not invalidate trial results. The data obtained during the trial will be treated under the applicable national and EU regulations.

Demographic and baseline characteristics will be presented for all patients. Discrete data will be summarized by frequencies and percentages. Continuous variables will be summarized using standard measures of central tendency and dispersion (mean and standard deviation or median and interquartile range).

The efficacy and safety analysis will be conducted on all patients undergoing surgery with cetuximab-IRDye800. A Hern’s Single-Stage Phase II design will be used. The study sample size has been calculated according to the efficacy endpoint. The efficacy analysis will be performed after the enrolment of 25 patients. The efficacy analysis will estimate the proportion of lung nodules correctly visualized by the NIR camera during surgery, with 90% confidence intervals (according to the 1-sided alpha error of 0.05). It is expected that cetuximab-IRDye800 will improve the proportion of NSCLC nodules detected intraoperatively from 40% (obtained employing white camera visualization) [7] to 70% by adding cetuximab-IRDye800. To conclude that the cetuximabIRDye800 is promising, the null hypothesis will be rejected if 15 or more patients out of 25 patients are correctly identified intraoperatively.

Toxicity will be recorded and classified according to the definitions of the NCI criteria “Common Terminology Criteria for Adverse Events (CTCAE),” version 5.0.

### Detailed procedure

The researcher will screen the candidate for a lung nodule excision procedure for suspected lung cancer. All the patients screened for suspected lung cancer and evaluated for lobectomy are studied in the preoperative period and undergo a total body CT scan, PET scan, and respiratory function tests as suggested in European and International guidelines (in addition, shuttle test and cardiopulmonary function test are performed if deemed necessary according to the respiratory function tests) and chest or brain MRI if needed for further clinical necessities. The patient’s clinical history, vital signs, clinical exams, blood tests, and radiological exams will be examined to assess inclusion and exclusion criteria. The patient signs the written informed consent during the screening visit and is enrolled in the study. The pregnancy test will be performed 1 to 2 days before the infusion of the study drug.

The drug infusion will be performed 2 to 5 days before the surgery: the patients will receive 100 mg of cetuximab intravenously over 30 min to differentiate between an infusion reaction to the unlabeled cetuximab and a cetuximab-IRDye800 reaction and to preload the hepatic EGFR, and a dose of 50 mg of cetuximab-IRDye800 over 30 min to 1 h [3]. The doses were determined based on previous findings by other authors, showing that 25 mg/m2 provided the optimal tumor-to-background ratio. EKGs (electrocardiograms) will be performed at screening, 30 min post-infusion of the unlabeled cetuximab loading dose, two h post-infusion of the cetuximab-IRDye800, and follow-up on day 30. After the drug infusion phase, patients will undergo VAT resections of the lung nodule (between days 2 and 5 post-infusion). During post-surgery hospital stays and after discharge to home, patients will be treated and followed up according to clinical practice.

Patients will be monitored for toxicity and compliance with a clinical assessment after discharge. Moreover, subjects will be instructed before participation on the importance of reporting any symptoms (new or worsening), and/or any physical changes throughout their participation in the study. Morbidity or toxicity will be assessed during and after drug infusion, during and after a hospital stay, and during and after the control visits. If subjects discontinue before study completion, they will be encouraged to return for safety evaluations.

Study drug will be discontinued for any of the following: Subject withdraws consent for the study, death attributed to study drug, serious systemic anaphylactic reaction (grade 3), or any serious rare reactions attributed to study drug.

## Discussion

### Expected results

We hypothesize that using cetuximabIRDye800 during minimally invasive surgical procedures for lung cancer could overcome the limitation demonstrated by ICG and the traditional localization strategies (e.g., coil, hook, dye intra-tumoral injection). We expect to validate the use of an optimal tracker that can be easily applied intraoperatively during minimally invasive lung surgical procedures, to define the optimal time window and the optimal dose of administration of the tracker, and to discover neoplastic localization in lymph nodes and lung parenchyma not predictable pre-operatively. In this sense, application in surgically treated pancreatic cancer and cancer Head and Neck has shown encouraging results [21, 22]. We performed the synthesis of the NIR tracker to verify the values of excitation/emission. Moreover, other groups have already published pre-clinical studies on the efficacy of anti-EGFR antibodies conjugated with NIR-Dye (including IRDye^®^ 800CW) in detecting lung cancer in both in-vitro and in-vivo settings [23]. Finally, several phase I-II clinical trials demonstrated that the cetuximab-IRDye800 could be safely administered to humans and that the adverse events of the labeled antibody (cetuximab-IRDye800) were consistent with the known toxicity profile of unlabeled cetuximab [24].

#### Ethics

As far as the ethics are concerned, cetuximab-IRDye800 was evaluated by several phases I-II clinical trials that demonstrated that it could be safely administered to humans and that its adverse events were consistent with the known toxicity profile of unlabeled cetuximab 24. All other exams and procedures are routine practice except for the control EKG (electrocardiogram) after and during study drug infusion. No major ethical concerns are present. The study will be covered by ad-hoc institutional insurance. All pre-clinical and clinical studies showed that the structure and the capability of the antibody to bind to EGFR, the clinical pharmacology, the clinical pharmacokinetic, and the clinical toxicology of cetuximab-800CW is therefore expected to be similar to that of unconjugated cetuximab. Moreover, since the total tracer dose (up to 50 mg flat dose) is much lower than doses given during the regular clinical application of cetuximab (up to 400 mg/m2, which translates to 680 mg for an average person), risks related to the drug infusion may be reduced in respect of the routine practice of cetuximab therapy. Moreover, if the drug infusion is successful in identifying lung nodules, lymph-nodal metastasis, or unexpected cancer localization (even just one of these outcomes), the patients enrolled in the study could benefit from more radical surgical procedure and a better cancer staging and consequently more adequate post-operative complementary therapy, that if submitted to the standard of care.

All authors have read and agreed to the published version of the manuscript.

## Data Availability

Data is provided within the manuscript.
